# Supplementary material for: Multi-stage models for the failure of complex systems, cascading disasters, and the onset of disease
Source: PLoS One. 2019 May 20;14(5):e0216422. doi: 10.1371/journal.pone.0216422 (PMC6527192; doi:10.1371/journal.pone.0216422)
Supplement: S1 Appendix — (PDF) [file pone.0216422.s001.pdf]

S1 Appendix

Derivation of Eq. 23 The solution of Eq. 16 can be written in terms of multiple definite integrals that are sometimes easier to evaluate or approximate than directly evaluating Eq. 16. It is equivalent to expressing the solution as multiple convolutions using Eq. 21, and changing variables appropriately. The equation is obtained by Taylor expanding all functions before taking their Laplace transform, inverting the Laplace transform of the product of all terms (which is easy to do for the powers of time that appear in a Taylor expansion), then using a product of Beta functions to factorise and re-sum the resulting expression. In mathematical notation,

$$\begin{aligned}\mathcal{L}^{-1}\left\{\Pi_{j=1}^{m}\mathcal{L}\left[f_j(t_j)\right]\right\} &= \mathcal{L}^{-1}\left\{\Pi_{j=1}^{m}\mathcal{L}\left[\sum_{n_j=0}^{\infty} f_{j,n_j}\frac{t^{n_j}}{n_j!}\right]\right\}\\ &= \mathcal{L}^{-1}\left\{\sum_{n_1=0}^{\infty}\cdots\sum_{n_m=0}^{\infty}\frac{f_{1,n_1}}{s^{n_1+1}}\cdots\frac{f_{m,n_m}}{s^{n_m+1}}\right\}\\ &= \sum_{n_1=0}^{\infty}\cdots\sum_{n_m=0}^{\infty} f_{1,n_1}\cdots f_{m,n_m}\frac{t^{-1+\sum_{i=1}^{m}(n_i+1)}}{\Gamma(\sum_{i=1}^{m}(n_i+1))}\end{aligned} \tag{1}$$

where $f_{i,n_j} = \partial^{n_j} f_i(t_i)/\partial t^{n_j}|_{t_i=0}$. Now noting that the Beta function has,

$$\int_0^1 u^{m-1}(1-u)^{n-1}du = \frac{\Gamma(m)\Gamma(n)}{\Gamma(m+n)} \tag{2}$$

we can write,

$$\frac{1}{\Gamma\left(\sum_{i=1}^{m}(n_i+1)\right)} = \frac{1}{\Gamma\left(\sum_{i=1}^{m-1}(n_i+1)\right)}\frac{1}{\Gamma\left(n_m+1\right)}\int_0^1 y_{m-1}^{-1+\sum_{i=1}^{m-1}(n_i+1)}(1-y_{m-1})^{n_m} \tag{3}$$

Repeatedly using this gives,

$$\begin{aligned}\frac{1}{\Gamma\left(\sum_{i=1}^{m}(n_i+1)\right)} &= \left(\Pi_{i=1}^{m}\frac{1}{\Gamma(n_i+1)}\right)\int_0^1 dy_1\ldots\int_0^1 dy_{m-1}y_1^0y_2^1y_3^2\ldots y_{m-1}^{m-2}\times\\ &(1-y_1)^{n_2}\ldots(1-y_{m-1})^{n_m}\times(y_1\ldots y_{m-1})^{n_1}(y_2\ldots y_{m-1})^{n_2}\ldots y_{m-1}^{n_m-1}\end{aligned} \tag{4}$$

Using Eq. 4 to replace $1/\Gamma\left(\sum_{i=1}^{m}(n_i+1)\right)$ in Eq. 1, and grouping terms,

$$\begin{aligned}\mathcal{L}^{-1}\left\{\Pi_{j=1}^{m}\mathcal{L}\left[f_j(t_j)\right]\right\} &= t^{-1+m}\int_0^1 dy_1\ldots\int_0^1 dy_{m-1}y_1^0y_2^1y_3^2\ldots y_{m-1}^{m-2}\times\\ &\left(\sum_{n_1=0}^{\infty} f_{1,n_1}\frac{t^{n_1}(y_1\ldots y_{m-1})^{n_1}}{\Gamma(n_1+1)}\right)\\ &\left(\sum_{n_1=0}^{\infty} f_{2,n_2}\frac{t^{n_2}(1-y_1)^{n_2}(y_2\ldots y_{m-1})^{n_2}}{\Gamma(n_2+1)}\right)\\ &\ldots\\ &\left(\sum_{n_m=0}^{\infty} f_{m,n_m}\frac{t^{n_m}(1-y_{m-1})^{n_m}}{\Gamma(n_m+1)}\right)\end{aligned} \tag{5}$$

The m Taylor series can now be re-summed to give,

$$\begin{aligned}\mathcal{L}^{-1}\left\{\Pi_{j=1}^{m}\mathcal{L}\left[f_j(t_j)\right]\right\} = t^{-1+m}\int_0^1 dy_1\ldots\int_0^1 dy_{m-1}y_1^0y_2^1y_3^2\ldots y_{m-1}^{m-2}\times\\ f_1\left(ty_1\ldots y_{m-1}\right)f_2\left(t(1-y_1)(y_2\ldots y_{m-1})\right)f_3\left(t(1-y_2)(y_3\ldots y_{m-1})\right)\ldots f_m\left(t(1-y_{m-1})\right)\end{aligned} \tag{6}$$

For example, taking $m=2$ gives,

$$f(t) = t\int_0^1 dy_1 f_1(ty_1)f_2(t(1-y_1)) \tag{7}$$

as we could have got from the convolution formula after a simple change of variables.

Eq. 6 might equivalently be regarded as a generalisation of a Schwinger/Feynman parameterisation, with,

$$
\begin{aligned}
\Pi_{j=1}^{m} g_j(s) \quad &= \int_0^1 dy_1 \ldots \int_0^1 dy_{m-1} y_1^0 y_2^1 y_3^2 \ldots y_{m-1}^{m-2} \times \\
&\mathcal{L}\left[t^{m-1}\mathcal{L}^{-1}\left\{g_1(s)\right\}(ty_1 \ldots y_{m-1})\right. \\
&\mathcal{L}^{-1}\left\{g_2(s)\right\}(t(1-y_1)y_2 \ldots y_{m-1}) \\
&\ldots \\
&\left.\mathcal{L}^{-1}\left\{g_m(s)\right\}(t(1-y_{m-1}))\right]
\end{aligned}
\tag{8}
$$

For example, taking $g_j(s) = 1/(s+a_j)^{p_j}$ and noting that $\mathcal{L}^{-1}\left\{1/(s+a_j)^{p_j}\right\} = t^{p_j-1}e^{-a_j t}/\Gamma(p_j)$, then we get,

$$
\begin{aligned}
\Pi_{j=1}^{m} \frac{1}{(s+a_j)^{p_j}} \quad &= \frac{\Gamma\left(\sum_{i=1}^{m} p_i\right)}{\Pi_{i=1}^{m}\Gamma(p_i)} \int_0^1 dy_1 \ldots \int_0^1 dy_{m-1} y_1^0 y_2^1 \ldots y_{m-1}^{n_m-1} \\
&(y_1 \ldots y_{m-1})^{p_1-1}\left((1-y_1)y_2 \ldots y_{m-1}\right)^{p_2-1} \ldots \\
&(y_{m-1}(1-y_{m-2}))^{p_{m-1}-1}(1-y_{m-1})^{p_m-1} \\
&\frac{1}{[s+(a_1 y_1 \ldots y_{m-1} + a_2(1-y_1)y_2 \ldots y_{m-1} + \cdots + a_m(1-y_{m-1})]^{\sum_{i=1}^{m} p_i}}
\end{aligned}
\tag{9}
$$

Taking $s = 0$, $m = 2$, and $p_j = 0$ for all j, gives the most well-known form, with [27],

$$
\frac{1}{a_1 a_2} = \int_0^1 \frac{dy_1}{(a_2 y_1 + (1-y_1)a_1)^2} \tag{10}
$$

The identity Eq. 9 can be confirmed by writing the denominator as $(A_m)^m$, with,

$$
A_m = (a_m(1-y_{m-1}) + y_{m-1}A_{m-1}(y_1, \ldots, y_{m-2})) \text{ and } A_1 = a_1 \tag{11}
$$

and integrating with respect to each of y_{m-1} to y_1 in turn. For example, using the substitution $u = y_{m-1}/(1+\alpha_{m-1}y_{m-1})$ with $\alpha_{m-1} = (A_{m-1} - a_m)/a_m$ and integrating between $u = 0$ and $u = 1/(1+\alpha_{m-1})$, the integrand becomes $(1/a_m)(1/{A_{m-1}}^m)$. Repeating this for y_{m-1} to y_1 confirms the identity.
